# Exploring the relationship between bearing extrusion and postoperative persistent pain in Oxford unicompartmental knee arthroplasty: A trajectory measurement study

**DOI:** 10.3389/fbioe.2022.965009

**Published:** 2022-09-29

**Authors:** Pengfei Wen, Qidong Zhang, Xiaowei Sun, Binfei Zhang, Tao Ma, Yumin Zhang

**Affiliations:** ^1^ Department of Joint Surgery, Honghui Hospital, Xi’an Jiaotong University, Xi’an, China; ^2^ Department of Orthopaedic Surgery, China-Japan Friendship Hospital, Beijing, China

**Keywords:** unicompartmental knee arthroplasty (UKA), bearing extrusion, postoperative knee pain, bearing movement trajectory, VAS

## Abstract

**Objective:** The aim of the study is to explore the relationship between the extrusion of the meniscus bearing and postoperative persistent pain of Oxford unicompartmental knee arthroplasty.

**Methods:** Patients undertaking Oxford UKA from January 2019 to June 2020 were retrospectively analyzed. Intraoperatively, the displacement and movement trajectory of the meniscus bearing was recorded by the specially designed gridding mold of the tibial component. The k-means clustering analysis was applied based on the incidence of postoperative persistent knee pain and the bearing extrusion distance. The intraoperative meniscus bearing movement trajectories were analyzed between the two groups and the patients’ clinical outcomes and radiographic assessments.

**Results:** The k-means clustering analysis indicated that the extrusion of the bearing of 5 mm was the grouping standard. There were 27 patients with 30 knees in the extrusion group and 58 patients with 68 knees in the non-extrusion group. The proportion of optimal bearing movement trajectories in the extrusion group was significantly lower than that in the non-extrusion group (*p* < 0.05). Postoperative persistent knee pain occurred in six cases (6.1%), with four and two cases in the extrusion and non-extrusion groups, respectively. The incidence of postoperative persistent knee pain in the extrusion group was higher than that of the non-extrusion group (*p* < 0.05). Radiographic assessment showed that the continuity of the femoral and tibial components in the extrusion group was greater than that in the non-extrusion group (*p* < 0.05). However, there were no differences in pre- and postoperative HKAA, the varus/valgus degree of both femoral and tibial components, and the flexion/extension angles of the femoral component, and the tibial slope also showed no statistical difference (*p* > 0.05).

**Conclusion:** For Oxford mobile-bearing UKA, the extrusion of meniscus bearing over 5 mm may increase the incidence of postoperative persistent knee pain, while the improvement of the bearing movement trajectory can effectively reduce this complication.

## 1 Introduction

Unicompartmental knee arthroplasty (UKA) is an effective option for treating anteromedial knee osteoarthritis (KOA) (Ateş et al., 2021). In recent years, due to faster postoperative recovery and better functional outcomes (Bayoumi et al., 2022), UKA has received increasing attention among orthopedic surgeons. Although UKA has obvious mini-invasive advantage compared to total knee arthroplasty (TKA), it has always been criticized for its complication and revision rate, which are reportedly higher than those of TKA (Kazarian et al., 2020; Di Martino et al., 2021). In a recent study, it was found that the lifetime revision risk of UKA was two times as that of TKA across all age periods (Tay et al., 2022).

Among the reasons for UKA revision, unexplained knee pain is one of the most common reasons and accounts for 14%–23% of all the reasons (Baker et al., 2012; van der List et al., 2016; Dyrhovden et al., 2017; Vasso et al., 2018). Different authors have different opinions on the origin of unexplained knee pain after UKA (Simpson et al., 2009; Lyu et al., 2021). Overhang of the tibial component was reported as a reason for knee pain after UKA (Chau et al., 2009; Gudena et al., 2013). During the physical examination for patients with knee pain after UKA, with the knee motion from flexion to extension, doctors can usually touch the extrusion of the meniscus bearing at the medial joint space and feel the tenderness at the same time. Therefore, the bearing extrusion may also lead to pain due to soft tissue impingement. This phenomenon has also been reported previously (Jung et al., 2009). However, the relationship between bearing extrusion and persistent pain after UKA is still unknown. The aim of this study is to explore the relationship between bearing extrusion and postoperative outcomes of Oxford UKA.

## 2 Materials and methods

We retrospectively reviewed all the cases of medial Oxford UKA from January 2019 to June 2020 in our hospital. The inclusion criteria included the following: 1) medial KOA; 2) no pain or tenderness in the lateral compartment; 3) no sign of infection in the surgical site and the whole body; 4) the varus deformity and flexion contracture were less than 15°; and 5) the function of the knee ligaments was good. Patients who fit either of the following conditions were excluded from the study: 1) KOA involved lateral compartment; 2) inflammation arthritis; and 3) incomplete clinical and radiological record. The study was approved by the ethics committee and was in accordance with the Declaration of Helsinki. All the patients included in the studies have signed the informed consent.

### 2.1 General information

In total, from January 2019 to June 2020, there were 162 UKAs of 137 patients being conducted in our hospital. Due to the COVID-19 epidemic, 64 UKAs of 52 patients were excluded for incomplete follow-up. Finally, the study enrolled 98 UKAs of 85 patients.

### 2.2 Surgical technique

All of the surgeries were performed by an experienced senior surgeon. The surgical procedure followed the surgical techniques of Oxford partial knee microplasty instrumentation (Zimmer). A thigh tourniquet was installed, and the thigh was positioned on the adjustable support, with about 30° hip flexion and knee freedom. With 90° knee flexion, a medial parapatellar incision was made from the medial edge of the patella to a 1.5 cm distal to the tibial plateau. The subcutaneous tissue was dissected, and the joint capsule was incised to expose the medial knee compartment. The size of the medial femoral condyle was measured with a femoral sizing spoon and connected with the tibial extramedullary positioning rod through a G-clamp. The tibial osteotomy direction was adjusted with a 7° posterior slope, fixed with a tibial saw guide, and the tibial plateau resection was performed. A hole was drilled in the intramedullary canal of the femur and an intramedullary positioning rod was inserted and then connected, and the femoral intramedullary rod was fixed with the femoral drill guide by a tuning fork. Two positioning holes on the distal medial femur condyle were drilled, the osteotomy device was inserted, and the posterior femoral condyle was resected. The flexion gap was measured and the femoral milling column was installed. After milling, the single-column prosthesis was installed. The gap tension at 20° and 90° of flexion was estimated according to the feeling of difficulty in inserting and pulling the inserts with bare hands. Depending on the gap tension, the femoral condyle was milled until the flexion and extension gaps are balanced. Osteophytes on the anterior and posterior femoral condyle are removed, the groove on the tibial plateau is cut with a toothbrush saw, and the trial implantation is installed. Then, the knee motion is checked and the flexion–extension gap balance is evaluated by pulling and inserting the insert again. Finally, the prosthesis is installed and the incision is closed without placing drainage.

### 2.3 Outcome measures

#### 2.3.1 Intraoperative measures

**FIGURE 1:**
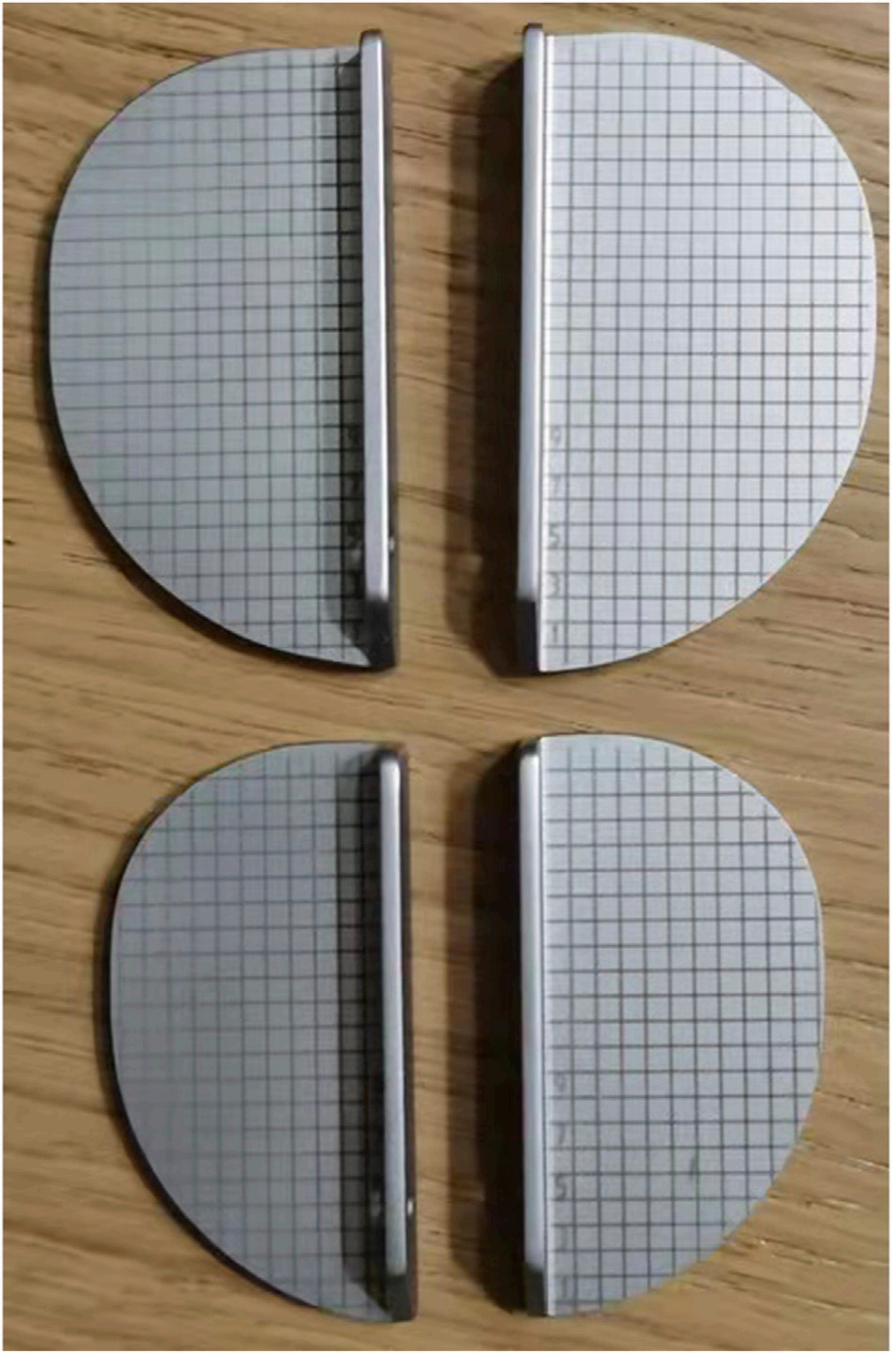
Gridding mold of the tibial component; the interval was 2 mm.

**FIGURE 2:**
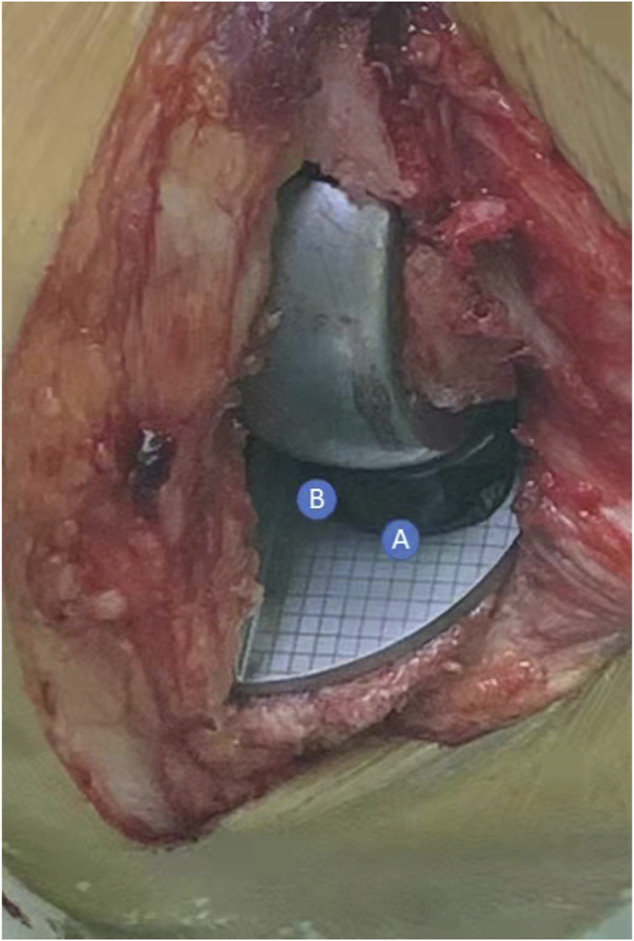
In 90° knee flexion, recording the coordinate of the anterior corner **(A point)** and anterolateral corner **(B point)** as the location of the meniscus bearing. The location of the meniscus bearing in other angles (90°, 60°, 45°, 30°, 20°, and 0°) was recorded in the same way and finally formed the movement trajectory.

**FIGURE 3:**
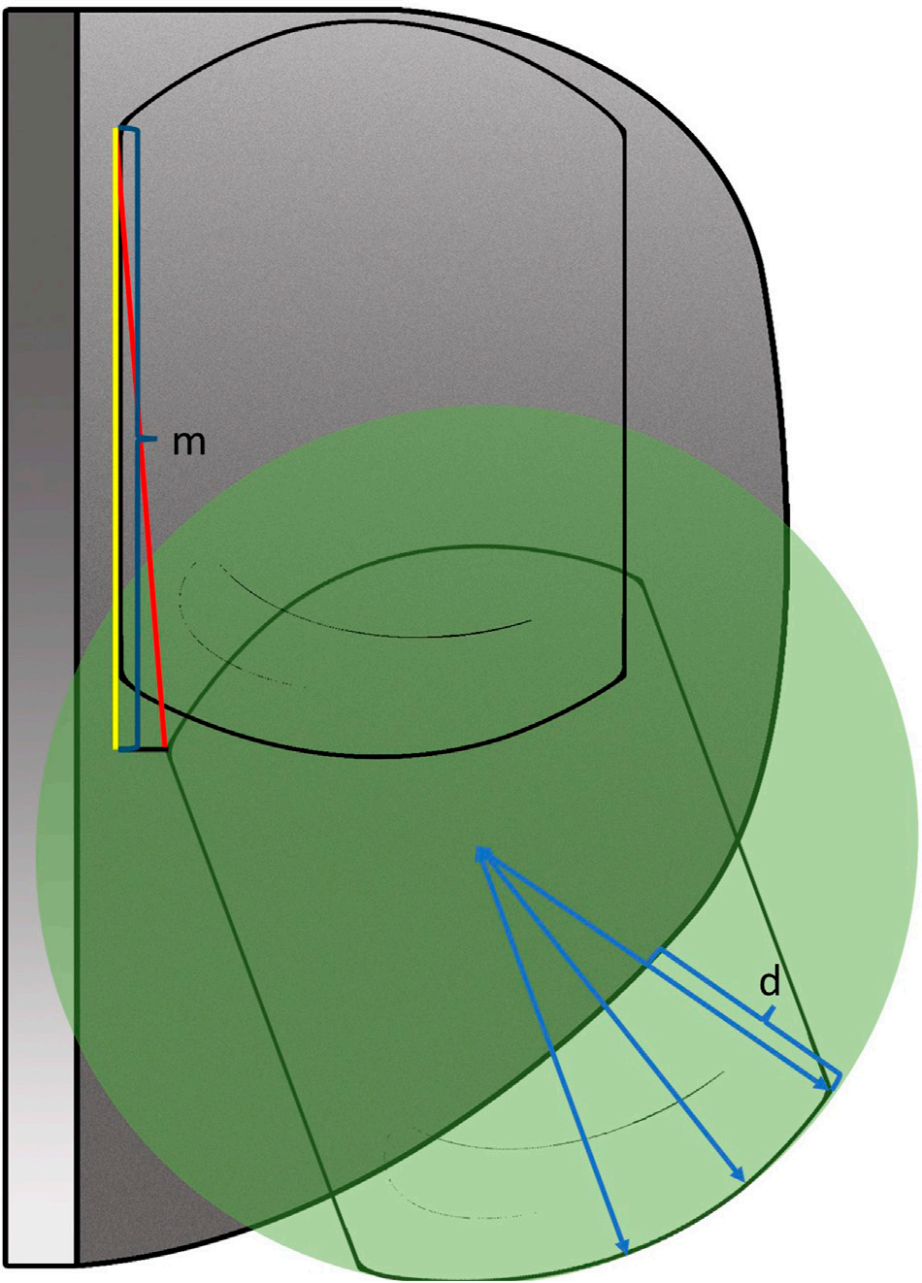
Diagram of the extrusion distance (d) and moving distance (m) of the meniscus bearing.

According to Kawaguchi’s method (Kawaguchi et al., 2019), we used a specially designed gridding mold of the tibial component (Figure 1) to record the displacement and the movement trajectory of the meniscus bearing mold. Before placing the real prosthesis, the location of the anterior corner and anterolateral corner of the meniscus bearing from flexion to full extension (90°, 60°, 45°, 30°, 20°, and 0°) was recorded (Figure 2). After placing the real prosthesis product, the distance of the bearing extrusion relative to the tibial component was measured. The extrusion distance was defined as the distance of the medial edge of meniscus bearing beyond the medial edge of the tibial component. It was measured through the line connecting the circle center of the anterior arc and the anteromedial end of the meniscus bearing (Figure 3).

#### 2.3.2 Clinical outcome assessment

The VAS score during walking was adopted for postoperative knee pain assessment. The clinical outcome was assessed by recording KSS clinical and functional scores of the preoperative and final follow-up.

#### 2.3.3 Radiological measures

**FIGURE 4:**
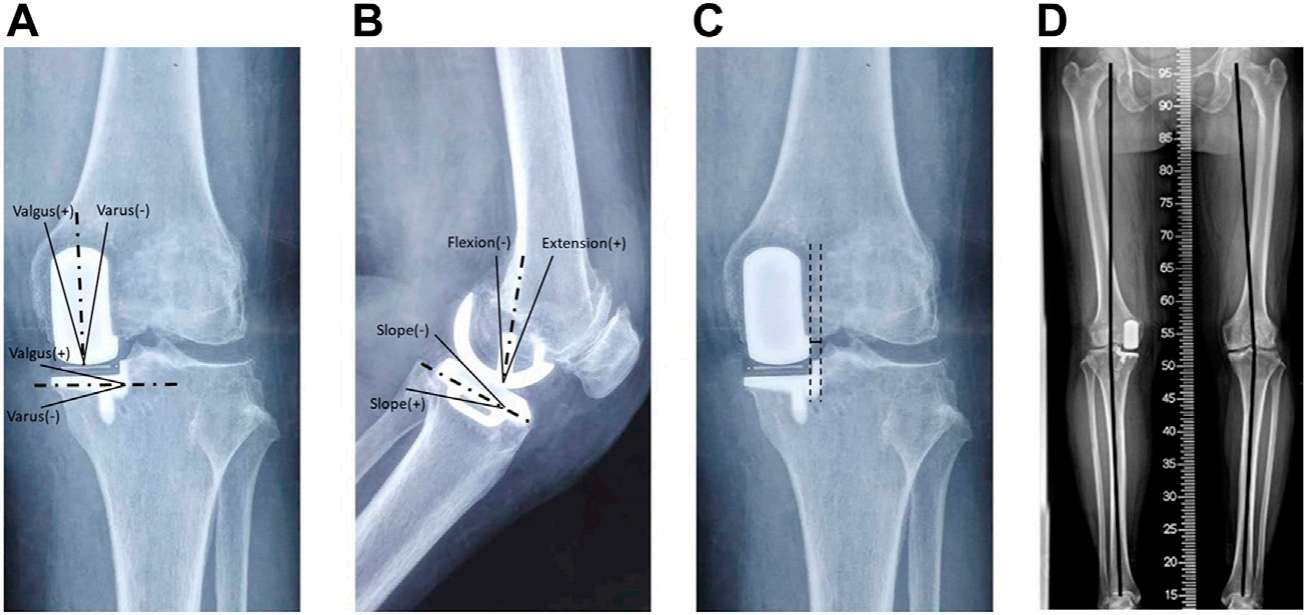
Radiological measures of the prosthesis after UKA. **(A)** Valgus/varus angle of femoral and tibial components on the antero-posterior view; **(B)** flexion/extension angle of the femoral component and slope of the tibial component; **(C)** adjacent degree between femoral and tibial components, a line is drawn vertical to the ground from the lateral edge of the femoral component, and the adjacent degree is measured as the horizontal distance between this line and the tibial vertical osteotomy line; **(D)** hip–knee–ankle angle (HKAA).

Patients commonly received preoperative and postoperative (within 5 days after UKA) X-ray examinations, including anteroposterior view, lateral view, Merchant view, and full-weight total length lower limb image. We measured the varus/valgus angle of the tibial and femoral components (Figure 4A), flexion/extension angle of the femoral component, and the slope of the tibial component (Figure 4B). We also measured the adjacent degree between the tibial and femoral components (Figure 4C). The hip–knee–ankle angle (HKAA) was measured on the full-weight bearing total length lower limb image (Figure 4D). Two authors independently measured these outcomes, and the mean value was adopted as the result for statistical analysis.

### 2.4 Statistics

SPSS 23.0 software was used for statistical analysis. K-means rapid clustering analysis was adopted for grouping (Sun et al., 2021a), and the final grouping was determined according to the clustering results and data characteristics. Measurement data were represented as (x̅ ± s). When the data were presented as normal distribution, the independent samples *t*-test was used to compare the two groups. Otherwise, the rank sum test was adopted. Fisher’s exact test was adopted for enumeration data. The Mann–Whitney *U* test was used for comparing the rank data between the two groups. Statistical significance of the difference was defined as *p* < 0.05.

## 3 Results

The k-means clustering was determined according to the postoperative persistent knee pain and extrusion distance of meniscus bearing, and the k value was set as 2. Finally, the 98 UKAs were divided into two groups; the sum of squared Euclidean distances from each point to the centroid between groups is equal. After grouping, we found the unique feature of group 1 (*n* = 30) and group 2 (*n* = 68) was extrusion distance ≥5 mm and extrusion distance <5 mm, respectively. Therefore, we defined the extrusion distance of 5 mm as the threshold value for meniscus bearing extrusion. As a result, the extrusion group consisted of 30 knees of 28 patients, and the non-extrusion group consisted of 68 knees of 58 patients. The baseline characteristics included age, sex, BMI, and follow-up time, and no statistical difference was found in these indexes (Table 1 **)**.

**TABLE 1:** Comparison of preoperative baseline characteristics of the two groups.

	Extrusion group	Non-extrusion group	*p*-value
	(*n* = 27)	(*n* = 58)	
Age (years)	66.85 ± 8.91	67.20 ± 9.63	0.721
Sex (M/F)	8/19	17/45	0.226
BMI(kg/m^2^)	26.26 ± 3.62	27.36 ± 4.05	0.186
Follow-up time (months)	27.35 ± 1.31	26.67 ± 3.24	0.443

### 3.1 Intraoperative measures

**FIGURE 5:**
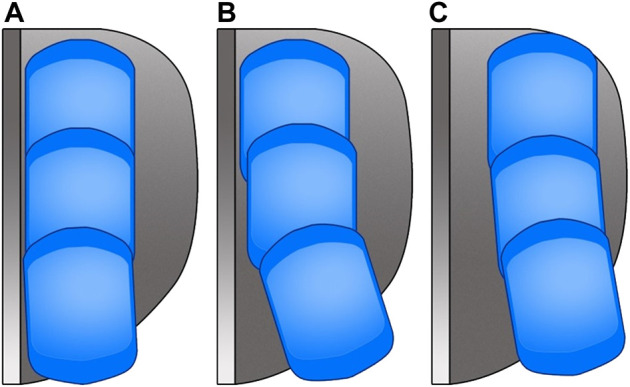
Diagram of the moving trajectory of the meniscus bearing. **(A)** Distance between the meniscus bearing and lateral wall of the tibial component is maintained stable within 2 mm during flexion/extension; **(B)** meniscus bearing is maintained close to the lateral wall of the tibial component during knee flexion and kept away from the lateral wall of the tibial component during knee extension, and the horizontal distance between the meniscus bearing and the lateral wall of the tibial component is over 2 mm in full extension; **(C)** meniscus bearing is consistently kept away from the lateral wall of the tibial component during knee flexion and knee extension. Type c was rarely seen in over study with only three cases.

The extrusion distance of the extrusion group was significantly greater than that of the non-extrusion group (*p* < 0.05), whereas no statistical significance was found in the moving distance between the two groups (*p* > 0.05). Furthermore, the movement trajectories of meniscus bearing were divided into three types. As shown in Figure 5, type a was defined as the optimal trajectory and types b and type c were defined as the non-optimal trajectory. The ratio of the optimal trajectory in the extrusion group was significantly lower than that in the non-extrusion group (*p* < 0.05) (Table 2).

**TABLE 2:** Comparison of the intraoperative measures on the movement trajectory of the meniscus bearing between the two groups.

	Extrusion group (*n* = 30)	Non-extrusion group (*n* = 68)	*p*-value
Extrusion distance (mm)	6.70 ± 1.02	2.97 ± 0.77	**<0.001**
Optimal trajectory (knees, %)	7 (23.33%)	66 (97.06%)	**<0.001**
Moving distance	8.57 ± 3.80	8.31 ± 4.14	0.571

### 3.2 Clinical outcomes

All the 98 UKAs of the two groups were carried out well, and all the incisions healed smoothly, with no delayed healing or infection event occurring. In total, six cases (6.1%) complained postoperative persistent knee pain, four cases belonged to the extrusion group, and two cases were in the non-extrusion group. Conservative drug or local block treatment was applied to these cases; the effect of pain release was acceptable but sometimes relapsed. However, due to the satisfied knee function, no patients required revision surgery. The rate of postoperative persistent knee pain in the extrusion group was significantly higher than that in the non-extrusion group (*p* < 0.05). No statistical difference was found in preoperative VAS score and pre- and postoperative KSS clinical and functional scores between the two groups (*p* > 0.05). But, statistical difference was found in postoperative VAS score between the two groups (*p* < 0.05) (Table 3).

**TABLE 3:** Comparison of the clinical outcomes between the two groups.

	Extrusion group (*n* = 30)	Non-extrusion group (*n* = 68)	*p*-value
Postoperative persist pain (knees, %)	5 (16.67%)	2 (2.94%)	**0.027**
KSS clinical score			
Preoperative	55.24 ± 6.31	57.49 ± 7.97	0.191
Last follow-up	89.93 ± 6.63	91.83 ± 5.22	0.138
*p*-value	**<0.001**	**<0.001**	
KSS function score			
Preoperative	55.37 ± 6.02	52.93 ± 9.44	0.215
Last follow-up	87.26 ± 11.08	90.00 ± 5.61	0.109
*p*-value	**<0.001**	**<0.001**	
VAS score			
Preoperative	7.81 ± 1.14	8.07 ± 1.10	0.312
Last follow-up	0.90 ± 1.52	0.35 ± 1.02	**0.039**
*p*-value	**<0.001**	**<0.001**	

### 3.3 Radiological outcomes

In all the cases of the two groups, the postoperative HKAA significantly increased than that before UKA (*p* < 0.05), whereas no statistical difference was observed in the pre- and postoperative HKAA between the two groups (*p* > 0.05). For postoperative varus/valgus angle in femoral and tibial components, sagittal flexion/extension angle in the femoral component, and the slope in the tibial component, no significant difference was presented between the two groups (*p* > 0.05). However, the adjacent degree between the tibial and femoral component was found to be significantly greater in the extrusion group than in the non-extrusion group (*p* < 0.05) (Table 4).

**TABLE 4:** Comparison of the radiological measures between the two groups.

	Extrusion group (*n* = 30)	Non-extrusion group (*n* = 68)	*p*-value
HKAA (°)			
Preoperative	171.89 ± 4.31	172.51 ± 3.69	0.347
Last follow-up	177.38 ± 3.11	177.86 ± 2.35	0.772
*p*-value	**<0.001**	**<0.001**	
Postoperative varus/valgus degree of the femoral component (°)	1.21 ± 4.53	0.48 ± 2.51	0.630
Postoperative flexion/extension degree of the femoral component (°)	9.65 ± 2.75	9.58 ± 3.11	0.758
Postoperative varus/valgus degree of the tibial component (°)	−2.02 ± 2.51	−1.06 ± 2.26	0.142
Postoperative tibial component slope (°)	8.39 ± 2.61	7.54 ± 1.37	0.197
Postoperative adjacent degree between tibial and femoral components	7.56 ± 3.21	4.82 ± 1.83	**<0.001**

## 4 Discussion

Persistent knee pain after UKA is a confusing complication for joint arthroplasty surgeons and a common reason for revision (Baker et al., 2012; Grosu et al., 2014; van der List et al., 2016). In recent years, many authors from different countries have reported this complication during the postoperative follow-up. Although the rate is quite different in different articles, the unhappy function and increasing revision rate resulting from this complication can lead to the declined satisfactory rates of UKA (van der List et al., 2016; Dyrhovden et al., 2017; Vasso et al., 2018). However, the reason for persistent knee pain after UKA has not yet reached a consensus, and some authors even directly named it as unexplained knee pain (Baker et al., 2012; Hama et al., 2015). Previous studies have reported some potential reasons for persistent knee pain after UKA, such as tibial component overhang (Simpson et al., 2009), overstuff of the medial knee compartment (Crawford et al., 2021), bursitis (Jung et al., 2009), and medial abrasion syndrome (Lyu et al., 2021). Meanwhile, according to the result in our study, the rate of postoperative persistent knee pain was 16.7% in the extrusion group and significantly higher than 2.94% in the extrusion group.

From the perspective of the prosthesis design of the Oxford UKA, slight meniscus bearing extrusion is common and unavoidable because the shape of the meniscus bearing is rectangle, whereas the tibial component has a distinct curving margin in the anterior edge. But in most situations, the meniscus bearing extrusion may not cause irritation to soft tissue around, which is quite different from tibial component overhang. Many studies have indicated that excessive coverage of the tibial component over 2 mm can cause impingement and stretching to medial collateral ligament (MCL) and may even further lead to serious complications like MCL loosening (Kim et al., 2014). The position of meniscus bearing extrusion is usually located in the anteromedial edge of the tibial plateau, and the local surrounding soft tissue is joint capsule and pes anserinus bursae. As they are not the main stabilization structures of the knee, slight meniscus bearing extrusion and soft tissue impingement may not lead to unhappy events. However, in previous literature, there were no criteria for the edge of meniscus bearing extrusion. Our study found meniscus bearing extrusion over 5 mm can lead to an increasing rate of persistent knee pain after UKA. Therefore, in medial Oxford UKA, the meniscus bearing extrusion should be controlled under 5 mm as possible.

**FIGURE 6:**
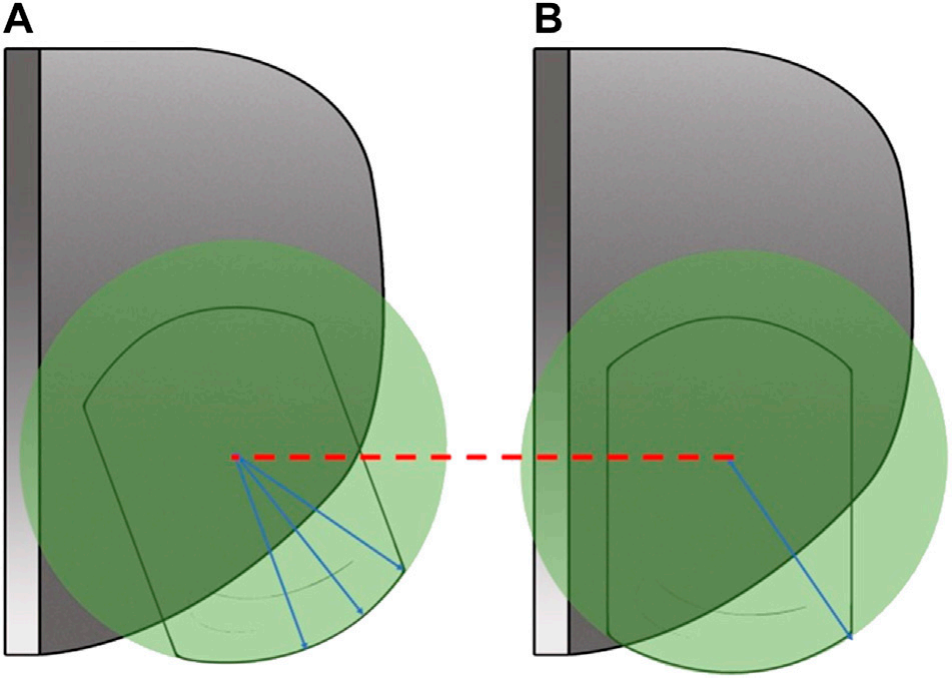
Compared to the optimal moving trajectory **(A)**, the non-optimal moving trajectory **(B)** may increase the extrusion distance and area of the meniscus bearing.

So, what caused meniscus bearing over-extrusion in medial Oxford UKA? Previous studies have found that the meniscus bearing was maintained parallel to the lateral wall of the tibial component during the knee flexion from 60° to 90° and kept away from the lateral wall of the tibial component during the knee flexion from 0° to 60° (Kamenaga et al., 2019; Kawaguchi et al., 2019). If the meniscus bearing separates a certain distance from the lateral wall of the tibial component, the possibility of bearing rotation is increasing, especially with the stimulation of external force. Under this condition, the limitation to the meniscus bearing from the femoral component is loosening, which is regarded as the mechanism for bearing dislocation (Clarius et al., 2010; Bae et al., 2020; Sun et al., 2021). The bearing dislocation can be regarded as an extreme condition of over-extrusion. Therefore, we hold that the mal-tracking of the meniscus bearing is the primary cause for meniscus bearing over-extrusion. As is shown in Figure 6, with the center of the meniscus bearing in same anteroposterior displacement, the non-optimal movement trajectory can lead to increasing extrusion distance and area. According to Kawaguchi’s classification to the movement trajectory of meniscus bearing, we analyzed and compared the trajectory between the two groups. The result showed that most trajectories in the extrusion group were non-optimal, and optimal trajectories occupied only 23.33%. By contrast, the rate of the optimal trajectory was up to 97.06% in the non-extrusion group, with statistical significance compared with the extrusion group (*p* < 0.05). Conversely, we further conducted a subgroup analysis for the 98 UKA cases, according to the optimal or non-optimal trajectory. The result showed the rate of postoperative persistent knee pain was only 2.74% in the optimal trajectory group, whereas it was up to 25% in the non-optimal trajectory group (*p* < 0.05). Moreover, the improper movement trajectory can also lead to rotation and dislocation of the meniscus bearing (Bae et al., 2020; Sun et al., 2021b). Therefore, we should pay sufficient attention to the movement trajectory of the meniscus bearing in Oxford UKA.

As the meniscus bearing is put between the femoral and tibial components to achieve the optimal bearing trajectory, the relative position between the femoral and tibial components is important. In detail, the key is the central aligning factor between the femoral and tibial components in the coronal plane. Previous studies also found over separation between the femoral and tibial components can increase the risk for bearing dislocation (Koh et al., 2016; Bae et al., 2020). In our study, we measured the adjacent degree to describe this meaning. The adjacent degree between the tibial and femoral components was found significantly greater in the extrusion group than in the non-extrusion group (*p* < 0.05). This indicated the central aligning between the femoral and tibial components in the extrusion group was superior to that of the non-extrusion group, which was in accordance to our theory. According to this concept, techniques for improving the movement trajectory of meniscus bearing in Oxford UKA have also been reported in recent years. Techniques like the kinematic alignment method with the extramedullary position technique of Zhang et al. (2020) and the modified tibial osteotomy technique introduced by Hiranaka et al. (2021) have all been proven to improve the movement trajectory of meniscus bearing.

There are also some limitations in our study. Due to the low rate of persistent knee pain after UKA, studies with larger samples or multi-center prospective studies are still required to get more accurate results and more convinced conclusions. In addition, the extrusion distance of meniscus bearing was measured manually using a disinfected ruler. The limited surgery time also cannot allow repeated measures by different observers. As a result, the system error is unavoidable.

## 5 Conclusion

In medial Oxford UKA, meniscus bearing extrusion over 5 mm may lead to an increasing rate of postoperative persistent knee pain. The over-extrusion may be due to the mal-tracking of the meniscus bearing. To reduce the rate of postoperative persistent knee pain, surgeons should pay sufficient attention to improve the movement trajectory of the meniscus bearing in Oxford UKA.

## Data Availability

The original contributions presented in the study are included in the article/Supplementary Material; further inquiries can be directed to the corresponding author.
